# Different Measures of Auditory and Visual Stroop Interference and Their Relationship to Speech Intelligibility in Noise

**DOI:** 10.3389/fpsyg.2017.00230

**Published:** 2017-03-17

**Authors:** Sarah Knight, Antje Heinrich

**Affiliations:** Medical Research Council Institute of Hearing Research, University of NottinghamNottingham, UK

**Keywords:** speech-in-noise, inhibition, aging, Stroop tasks, scoring

## Abstract

Inhibition—the ability to suppress goal-irrelevant information—is thought to be an important cognitive skill in many situations, including speech-in-noise (SiN) perception. One way to measure inhibition is by means of Stroop tasks, in which one stimulus dimension must be named while a second, more prepotent dimension is ignored. The to-be-ignored dimension may be relevant or irrelevant to the target dimension, and the inhibition measure—Stroop interference (SI)—is calculated as the reaction time difference between the relevant and irrelevant conditions. Both SiN perception and inhibition are suggested to worsen with age, yet attempts to connect age-related declines in these two abilities have produced mixed results. We suggest that the inconsistencies between studies may be due to methodological issues surrounding the use of Stroop tasks. First, the relationship between SI and SiN perception may differ depending on the modality of the Stroop task; second, the traditional SI measure may not account for generalized slowing or sensory declines, and thus may not provide a pure interference measure. We investigated both claims in a group of 50 older adults, who performed two Stroop tasks (visual and auditory) and two SiN perception tasks. For each Stroop task, we calculated interference scores using both the traditional difference measure and methods designed to address its various problems, and compared the ability of these different scoring methods to predict SiN performance, alone and in combination with hearing sensitivity. Results from the two Stroop tasks were uncorrelated and had different relationships to SiN perception. Changing the scoring method altered the nature of the predictive relationship between Stroop scores and SiN perception, which was additionally influenced by hearing sensitivity. These findings raise questions about the extent to which different Stroop tasks and/or scoring methods measure the same aspect of cognition. They also highlight the importance of considering additional variables such as hearing ability when analyzing cognitive variables.

## Introduction

Inhibition—the ability to suppress goal-irrelevant information (MacLeod, 1991)—is thought to be important in many situations. One of these situations is speech-in-noise (SiN) perception, in which listeners aim to focus on the foreground (target speech) and ignore the background (distractor) sound. The ability to inhibit irrelevant information has been suggested to worsen with age (Hasher and Zacks, 1988), with implications across a variety of cognitive domains including language, memory, and attention (Stoltzfus et al., 1996; Burke, 1997). This cognitive decline has potential consequences for everyday activities such as reading and text comprehension (Dywan and Murphy, 1996) and even engaging in appropriate social behavior (von Hippel, 2007). The ability to understand speech-in-noise is also observed to worsen with age, affecting the ability to hold conversations and engage in social activities (CHABA, 1988). Given the suggested importance of inhibition for SiN perception, researchers have begun to ask whether or not age-related declines in inhibition could account, at least in part, for the observed difficulties older adults have when listening in noisy environments. However, answering this question has been made difficult by the fact that it is not clear what role modality plays in the measurement of inhibition (whether or not inhibition tasks in different modalities measure the same underlying ability) and whether the standard scoring method adequately accounts for other, unconnected, age-related changes.

In the following section we introduce two types of Stroop task, a paradigm commonly used to assess inhibitory abilities and the focus of this study. We first explain the nature of Stroop tasks, and discuss the effect perceptual modality has on task outcomes. Next, we explore the effect of age-related changes on Stroop interference and consider potential underlying mechanisms. Finally, we discuss how the most common outcome measure of Stroop interference, reaction times (RTs), may relate to strength of inhibition, and propose that trials which are responded to more slowly may not only represent inhibition more accurately than trials responded to more quickly but may also better reveal differential levels of inhibition between participants. We then turn to speech-in-noise perception, and discuss the possible role of inhibition in SiN perception. In particular, we focus on the role inhibition plays during lexical access, a key element of speech perception, and consider how changes across the lifespan in lexical access might indicate age-related changes in inhibition. Finally, we discuss the results obtained from existing studies designed to test the relationship between inhibition and SiN perception, and suggest some reasons why these discrepancies might arise.

### Stroop tasks

One common means of assessing inhibition is by using variants of the Stroop task (Stroop, 1935). In the traditional visual color-word Stroop task (ibid.), participants are required to name the ink color of a string of letters, irrespective of the letters themselves. The string of letters can be either meaningless (e.g., XXXX)—the neutral condition—or can form a conflicting color word (e.g., BLUE printed in red)—the incongruent condition. Since word reading is a more prepotent response than color naming in this situation (Melara and Algom, 2003), word naming has the potential to interfere with color naming. In order to prevent this interference, participants must attempt to inhibit, or suppress, the incongruent word. The difference in reaction time (RT) between color naming in the neutral condition and color naming in the incongruent condition is taken as a measure of inhibitory ability, and termed Stroop interference (SI). Besides the traditional visual paradigm, auditory versions of the Stroop task have also been successfully used (e.g., Green and Barber, 1981; Morgan and Brandt, 1989). In auditory Stroop tasks, participants are required to respond as quickly as possible to some perceptual feature of a word (e.g., speaker gender, voice pitch, stimulus location) while ignoring the semantic information, which can be either irrelevant (e.g., “cat”) or conflicting (e.g., “man” spoken by a woman, “low” in a high-pitched voice, “right” heard in the left ear). Again, SI is typically obtained by calculating the difference in reaction time between feature naming with irrelevant semantic content and feature naming with an incongruent semantic distractor.

#### Stroop tasks across modalities

The visual and auditory versions of the Stroop task are generally assumed to tap the same underlying domain-general inhibitory ability; however, the relationship between the two measures and the extent to which this assumption is true remains unclear. On the one hand, there is evidence to suggest that carefully-matched Stroop tasks presented across different modalities do probe shared inhibitory processes, producing similar patterns of neural activation and correlated behavioral responses (Roberts and Hall, 2008). On the other hand, it has been shown that, even within the same modality, measures of inhibition that are not so closely matched do not correlate within individuals, suggesting either that there is no single inhibitory function supporting performance across different tasks and/or that task-specific demands determine individual differences more strongly than general inhibitory abilities (Shilling et al., 2002). This suggests that any two inhibition tasks, either within or across modalities, are unlikely to be comparable unless they have been deliberately matched, and in particular that an auditory Stroop task cannot automatically be assumed to be an alternative way of measuring the same ability tapped by a given visual Stroop task. In the current study we will address the question of the relationship between visual and auditory versions of the Stroop task by comparing scores from the same participants on an auditory and a visual Stroop task, both deliberately chosen to meet certain criteria.

#### Age-related declines in stroop performance

When calculated in the traditional way, SI (Stroop interference) on both visual and auditory tasks is generally observed to increase with age, implying a worse performance on the incongruent Stroop task compared to the neutral condition and—hence—poorer inhibition. However, it has long been recognized that no task is ever a “pure” measure of a given cognitive function, but instead includes other, additional processes—something referred to as the “impurity principle” (Surprenant and Neath, 2009). In the case of the Stroop task, it has been suggested that these age-related increases in SI could be due, at least in part, to just such additional processes; that is, that there are potential confounds with non-inhibitory factors created by the methods typically used to calculate SI (Ben-David and Schneider, 2009)—and that methods should be used which account for these factors.

One of these confounds is generalized age-related slowing. In the traditional SI measure, inhibition is represented by the absolute difference in time taken to name the background color between conditions with and without a distracting color word. A change in the speed of processing would slow performance on all tasks by the same factor (Cerella and Hale, 1994; Verhaeghen and Cerella, 2002), leading to a proportional increase of RTs in the incongruent and neutral conditions; this would result in a larger absolute difference between RTs in the two conditions, and thus a larger SI (Shilling et al., 2002; Ben-David and Schneider, 2009). Crucially, in such a case the increased SI does not necessarily represent any decline in inhibitory ability, but a change in processing speed. One way to address this issue is to use a method for calculating Stroop scores which accounts for, or factors out, changes in overall processing speed. For example, it is possible to use normalized scores, in which the RT in the incongruent condition is divided by the RT in the neutral condition, thus removing any changes in SI caused by proportional RT increases in both conditions. This is further discussed in “Calculating Visual Stroop scores” in the Materials and Methods section below.

While a generalized slowing of processing speed is expected to affect Stroop tasks across different modalities in similar ways, the confounding effects of sensory change will be specific to the perceptual domain of any given Stroop task. For visually presented Stroop tasks, such confounding effects may be particularly critical when they adversely affect the RT of the incongruent condition. If we accept the proposal of Melara and Algom (2003) that the Stroop interference effect arises due to a failure to inhibit the more rapidly accessed printed word until access to the incongruent color name is achieved, then changes in color vision may make access to the color word slower and/or more difficult, thereby increasing reaction times during color naming (Ben-David and Schneider, 2010). Such changes could be brought about by age-related yellowing of the lens and a loss of photo receptors (Werner and Steele, 1988; Anstey et al., 2002). These age-related changes in color vision do not affect word reading (Salthouse and Meinz, 1995), the speed of which remains largely unchanged with age provided the words are sufficiently legible (Akutsu et al., 1991). As a result, the difference between the time taken to read incongruent words and to name ink colors will be much greater for individuals with an age-related decline in color vision than for those with better color vision (i.e., younger adults). Melara and Algom (2003) characterized this discrepancy between color naming speed and reading speed as the “Dimensional Imbalance,” or DI. Having a larger DI—that is, a greater discrepancy in processing time between reading and color naming—puts individuals at an increased risk of a failure of inhibition (as expressed in larger SIs), since participants have to suppress the irrelevant word for longer. In this case, then, increased SI scores may reflect a combination of reduced inhibitory control and an increased likelihood of inhibitory failure caused by differences in processing speed for words as opposed to colors (i.e., a large DI). One way to address this issue is to use a method for calculating Stroop scores which accounts for, or factors out, differences in DI. For example, it is possible to regress RTs in the incongruent condition on DI scores, and then use the residuals as a measure of Stroop interference. This is further discussed in “Calculating Visual Stroop scores” in the Materials and Methods section below.

In the current study we will examine the effect of general age-related slowing and age-related sensory changes by comparing alternative scoring methods that capture age-related changes in inhibitory ability to different extents.

#### RT distributions in stroop tasks

In addition to questions of how to appropriately capture the differential age trajectories of the processes contributing to the overall effect, there is a further issue with the way in which Stroop scores are traditionally calculated, namely that they usually use an average score over all trials. If it is true (e.g., Ridderinkhof et al., 2004) that the strength of inhibition depends on the overall processing time, with the slowest responses allowing more time for inhibition to build up, then differences in inhibitory ability are likely to be most evident during those trials with the longest reaction times. That is, trials with longer reaction times will be more informative when assessing inhibitory differences than trials with shorter reaction times, since the gap between those with good inhibition and those with poor inhibition will be at its most pronounced. In averaging over all trials, the traditional SI measure may blur crucial information by mixing outcomes from some informative (slow) trials with outcomes from many uninformative (fast) trials. In the second part of the paper we will examine this hypothesis by investigating the differing extent of Stroop interference for slow and fast trials.

### Speech-in-noise perception and inhibition

Research into SiN perception difficulties in older adults has revealed that only some of these difficulties can be accounted for by hearing loss, and that other abilities must play a role (Schneider and Pichora-Fuller, 2000; Wingfield and Tun, 2007). One of those abilities is cognition, which must be examined alongside hearing loss in order to better explain age-related difficulties (Akeroyd, 2008). Cognition is not a unitary construct, and has many different components. The exact number and nature of the cognitive components varies across different cognitive models; however, inhibition is generally identified as a core ability (e.g., Conway and Engle, 1994; Friedman and Miyake, 2004; Baddeley, 2012; Diamond, 2013). Two potential ways in which inhibition may affect SiN perception have been suggested. First, poor inhibition may increase susceptibility to background noise during SiN listening (Janse, 2012). This implies not only that those with poor inhibition will perform worse on SiN tasks than those with good inhibition, but also that their difficulties may increase disproportionately as the signal-to-noise ratio (SNR) becomes more adverse. Second, it is suggested that poor inhibition may make it harder for listeners to successfully select the target during lexical access (Sommers and Danielson, 1999).

#### Lexical access and inhibition

One way to conceptualize lexical access is in terms of the Neighborhood Activation Model (NAM) (Luce and Pisoni, 1998). The NAM proposes that items in the mental lexicon are organized into similarity neighborhoods, defined as all words that can be created from a target item by adding, deleting or substituting a single phoneme. Any given target word will activate both the target and, to varying degrees, its surrounding neighborhood, which may be large (dense) or small (sparse); furthermore, words which are more commonly encountered (have a high frequency of occurrence) will be activated more strongly than those less commonly encountered. Words are therefore classified as “lexically easy” if they have a high word frequency and relatively sparse neighborhoods, and as “lexically hard” if they have a low word frequency and relatively dense neighborhoods (e.g., Taler et al., 2010). It is assumed that inhibition plays a larger role in the perception of lexically hard words than easy words (Sommers and Danielson, 1999). It is therefore expected not only that listeners will be less likely to correctly identify lexically hard words than lexically easy words, but also that individual differences in inhibition will relate more closely to the perception of lexically hard words than lexically easy words. The first prediction has been borne out experimentally in studies with normal-hearing adults (Sommers and Danielson, 1999; Taler et al., 2010; Helfer and Jesse, 2015), children (Eisenberg et al., 2002), cochlear implant users (Kaiser et al., 2003; Bierer et al., 2016) and native and non-native speakers (Bradlow and Pisoni, 1999); the second prediction has also received some experimental support (Sommers and Danielson, 1999; Taler et al., 2010) and will be further tested in the current study.

Lexical access can also be affected by the semantic context provided by the words preceding the target: a certain semantic context can markedly increase the likelihood that a given word will occur. It is commonly found that recognition is better for words in semantically meaningful sentences than words in isolation (Miller et al., 1951; Nittrouer and Boothroyd, 1990), and for items in sentences with higher as opposed to lower semantic predictability (Bilger et al., 1984). These findings can also be explained in terms of the NAM: as semantic information builds over the course of a sentence, it increases activation levels for contextually consistent words (Sommers and Danielson, 1999).

The phenomenon of retrieval-induced forgetting has also been suggested by some researchers (e.g., Anderson et al., 1994; Aslan and Bäuml, 2011) as evidence for the role of active inhibition in lexical access [however, see e.g., MacLeod et al. (2003) and Williams and Zacks (2001) for alternative interpretations]. Retrieval-induced forgetting refers to a situation in which recall for verbal material suffers when related material (e.g., a member of the same category) has earlier been cued and correctly recalled. This suggests that inhibitory processes suppress relevant but uncued material during the initial recall phase, leading to poorer recall for that same material later.

#### Age-related changes in inhibition and lexical access

The fact that effects of lexical difficulty and semantic context on word recognition vary through the lifespan has been taken as indicating age-related changes in inhibition. For example, the finding that identification of isolated lexically hard words declined with age, while performance for isolated lexically easy words was comparable for younger and older listeners, was interpreted by Sommers (1996) as reflecting an age-related decline in inhibitory control: since competing words from the target's neighborhood have to be suppressed or inhibited for successful word identification, poorer inhibition would reduce the ability to perform the required suppression of competing words and hence result in lower performance for lexically hard words. Results from the audiovisual (AV) domain have been interpreted in a similar vein: the finding that older adults were disproportionately poorer at identifying words with dense audiovisual neighborhoods was taken as indicating an age-related decline in inhibition (Dey and Sommers, 2015); this hypothesis was supported by the fact that Stroop scores predicted AV word recognition in older, but not younger, adults. Finally, Sommers and Danielson (1999) attribute Pichora-Fuller et al.'s (1995) finding that older listeners benefitted more from the addition of semantic context than younger listeners to higher activation of contextually consistent words amongst older listeners due to increased linguistic experience.

However, it is important to note that several studies have failed to show a relationship between inhibitory abilities and SiN perception (Gilbert et al., 2013; Helfer and Freyman, 2014). It is unclear why these discrepancies arose, but one possibility is that the differences were due, at least in part, to the methodological issues described above. Although all of these studies used Stroop tasks to assess inhibition, they differed in the modality of the task used (auditory vs. visual), and in the way in which Stroop interference was calculated. In particular, some used traditional SI scores, which as discussed above may be subject to confounds with generalized slowing and/or sensory decline, while others used adjusted scoring systems that may have accounted for slowing, poor color vision or both. In order to better understand the relationship between inhibition, SI scores and SiN perception, and to investigate how the predictive relationship between SI scores and SiN perception changes depending on whether or not possible confounds in the SI measures have been taken into account, we assessed the predictive value for SiN perception of SI measures derived from an auditory and a visual Stroop task using scoring methods that did or did not account for possible age-related confounds. If the power of Stroop scores to predict SiN perception is based on their ability to measure inhibition, then a purer inhibitory measure free from age-related confounds should improve prediction. However, Stroop scores may primarily measure more general age-related changes, such as generalized slowing and sensory declines. Since generalized slowing will affect performance across a range of tasks, and sensory declines are likely to be shared across the visual and auditory domains (Lindenberger and Baltes, 1994), the predictive relationship between Stroop scores and SiN perception may be based more strongly on these age-related changes than on inhibition. If this is the case, then the traditional, unadjusted SI measures should prove more useful in predicting SiN performance.

## Hypotheses

### Different scoring systems

H1: Scoring methods can be devised that do or do not take age-related changes in processing speed and sensory decline (i.e., poorer color vision) into account. If non-inhibitory age-related changes are independent contributors to Stroop scores alongside inhibitory ability (Melara and Algom, 2003), we would expect a low correlation between traditional scores, which do not account for these age-related changes, and the new scores, which do.

H2: Stroop scores can be calculated across all trials, or only across trials which are responded to particularly slowly or quickly. We expect the size of the Stroop effect to be larger on average for the slower trials than the faster trials, since a proportional slowing of both longer (incongruent trial) and shorter (neutral trial) RTs leads to a larger differences between the two overall when using the traditional calculation method. If it is true that differences in inhibitory ability are more in evidence when participants take longer to respond (Ridderinkhof et al., 2004), then we also expect to see greater variation in individual Stroop effects when examining slower trials as opposed to faster trials.

### Visual vs. auditory tasks

H3: The results from the visual and auditory Stroop tasks will be broadly comparable, assuming that (a) inhibition is a modality-independent general cognitive ability, (b) inhibition influences individual performance to a greater extent than do task-specific demands, and (c) the two types of task are tapping into the same ability. If this is not the case, this raises questions about the extent to which the two tasks measure the same aspect of cognition.

### Relationship to SiN tasks

H4: Based on previous studies (Sommers and Danielson, 1999; Janse, 2012) we predict larger Stroop interference (SI) scores to be predictive of worse performance on SiN tasks—that is, a negative relationship between SI scores and SiN scores. If SI scores provide a genuine measure of inhibitory ability, then this relationship should be particularly strong when the SiN stimuli demand high levels of inhibition: at lower (less favorable) SNRs, when sentential context is lacking (i.e., when targets are isolated words), when target words have a low word frequency and/or high neighborhood density, or when semantic context does not aid inference (i.e., when targets appear in low-predictability sentences). It is possible that these effects may be particularly pronounced for those with poorer hearing sensitivity (Helfer and Jesse, 2015).

H5: If the relationship between SI scores and SiN perception is partially driven by shared sensory decline, we might expect the predictive power of Stroop interference for speech perception to decrease once sensory decline is taken into account. If, on the other hand, it is the inhibition component of the Stroop task that drives the relationship with speech perception, then a purer measure less affected by sensory change might improve the association between the two measures.

H6: Based on previous studies suggesting that differences in inhibitory ability are more in evidence when participants take longer to respond (Ridderinkhof et al., 2004), we expect Stroop scores derived from slower trials to be better predictors of SiN perception than scores derived from faster trials or averages across all trials.

## Materials and methods

### Participants

Participants were 50 adults aged over 60 (mean: 69.5 years, SD: 6.4, range = 61–86) with mild hearing loss. A sample size of *N* = 50 allowed for the detection of a medium-sized effect (*r* = 0.35) at alpha (two-tailed) = 0.05 with a probability of 80%. This was deemed sufficient given that the most closely related previous studies (Sommers and Danielson, 1999; Janse, 2012) typically show medium-to-large effect size correlations. Exclusion criteria were hearing aid use and non-native English language status. This study was carried out in accordance with the recommendations of the University of Nottingham's Code of Research Conduct and Research Ethics, with written informed consent from all participants. All participants gave written informed consent in accordance with the Declaration of Helsinki. The protocol was approved by the University of Nottingham's School of Psychology Ethics Committee (ref. 464).

Visual accuracy was assessed using a Landolt C Chart, and color vision was tested using the card version of the City University color Vision Test. All participants were able to successfully read a full line of optotypes on the Landolt C Chart at a logMAR value of at least 0.3, with the majority (34) able to read a full line at between −0.1 and 0.1 logMAR. Four participants failed the color Vision Test, and the same group also verbally reported color blindness; these participants were excluded from the visual Stroop task. No other participant reported any difficulty in reading the test materials for the visual Stroop task. Two participants were excluded from the auditory Stroop task due to technical failure. Additionally, all participants were screened for mild cognitive impairment (MCI) using the Montreal Cognitive Assessment (MoCA) (mean: 27.86; SD: 1.95).

The reported results are part of a larger study into cognitive contributions to speech perception in older adults. Unreported results do not relate to the topics discussed in this paper.

### Auditory measures

**Figure 1:**
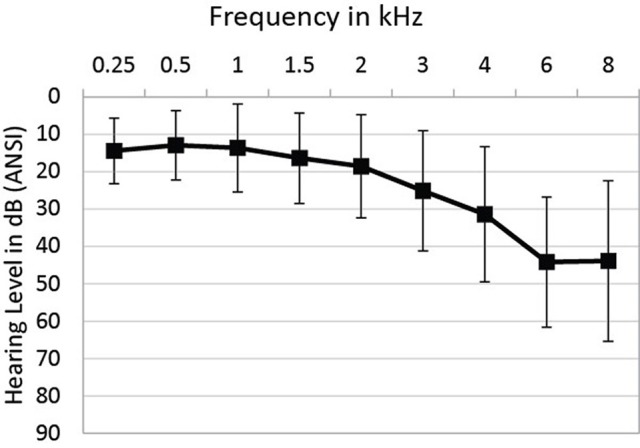
**Mean PTA thresholds as a function of frequency**. Bars indicate +/−1 standard deviation.

Pure-tone air-conduction thresholds (PTA) were collected for nine frequencies between 0.25 and 8 kHz for each ear, following the procedure recommended by the British Society of Audiology (British Society of Audiology, 2011) using an Interacoustics Audiometer AT235 (Interacoustics, Middelfart, Denmark) and TDH39P headphones (Telephonics, Farmingdale, NY, USA). Mean thresholds as a function of frequency are presented in Figure 1. As this figure shows, there was considerable variability between participants in terms of hearing sensitivity, particularly at the higher frequencies.

Speech reception thresholds (SRT) were obtained using 30 sentences from the Adaptive Sentence List (Macleod and Summerfield, 1990). Sentences were initially presented at 60 dB SPL, with a one-down-one-up procedure and step sizes of 10 dB down, then 5 dB up for the first reversal; the remainder of the trials used a three-down-one-up procedure with a step size of 2 dB. The last two reversals were averaged to determine the 79% accuracy point (Levitt, 1971). Based on this, all auditory stimuli used throughout the study, including the auditory Stroop stimuli, were presented at 30 dB SL—that is, 30 dB above each participant's individual threshold. This procedure was used to partially control for differences in intelligibility in quiet due to the considerable range in participants' hearing sensitivity.

### Stroop tasks

In the visual Stroop task, modeled after Janse (2012), participants were presented with grids formed of 48 boxes in an 8 × 6 arrangement. There were three types of grid: (i) a reading grid, consisting of white boxes containing black color words; (ii) a control grid, consisting of colored boxes containing the string “XXXX” in black; (iii) an interference grid, consisting of colored boxes containing mismatched color words in black. The colors used were red, blue, green and brown. Using relatively large boxes of color instead of font color maximized the opportunity for older participants to clearly see the colors. The distractor words were printed in black and displayed in each box using 20 pt Calibri font. In order to ensure best possible visibility the light in the test room was always at least 880 lux and was set in such a way that each participant could optimally see colors and text without experiencing glare. For (i), the task was to read the words aloud as quickly and accurately as possible. For (ii) and (iii), the task was to name the background color of the boxes as quickly and accurately as possible. There was a short practice session for each of the 3 tasks. Participants saw two versions of each grid. The total time taken to complete each grid was timed by the experimenter using a stopwatch, and overall scores for each grid type were calculated by averaging the two times obtained. Some participants made errors on the interference grid. In these cases, no penalty was applied if they corrected their mistake. Uncorrected mistakes were penalized by calculating the participant's average time per item on the interference grid in question, then adding this duration to their total grid time once for each mistake. The times for the reading and control grids represented error-free performance for all participants.

In the auditory Stroop task, modeled after Sommers and Danielson (1999), participants heard two male and two female speakers, and were required to respond as quickly and accurately as possible to the gender of the speaker. Any given trial consisted of one of three words: “mother,” “father” or “person.” These words could therefore be congruent with gender (e.g., female + “mother”), incongruent with gender (e.g., male + “mother”) or neutral (“person”). RTs for gender decisions were obtained via button presses. Participants always used their self-reported dominant hand to respond, and returned their hand to the rest position in front of the button box after the end of each trial. For each trial, the RT was measured from the onset of the sound file; however, the recordings had been trimmed so that, for the words “father” and “person,” voicing started at a similar point in all files (around 13 ms after onset for “father,” and around 7 ms after onset for “person”). For “mother,” voicing was considered to start early enough that the point of vowel onset was not meaningfully different between any of the four recordings. The location (left/right) of the buttons corresponding to “female” and “male” were swapped for half of the participants. Participants received a short practice session containing all three conditions before the start of the task.

#### Calculating visual stroop scores

The Stroop interference measure (SI) traditionally used in the literature (MacLeod, 1991) is calculated as follows:

(1) $$\begin{array}{r} \text{vS}{\text{I}}_{\text{raw}}=\text{Ci}-\text{Cn} \end{array}$$

One problem with using the traditional SI measure as an estimate of inhibition in older adults is that there can be age-related changes in general processing speed (Ben-David and Schneider, 2009). This would be expected to slow performance on incongruent (Ci) and neutral (Cn) trials by the same factor, leading to different absolute increases—which in turn lead to larger SI values when the difference between the two conditions is calculated. A possible way to account for this age-related change and minimize its effect on interference estimates is to use a normalized measure of Stroop interference. This can be calculated as follows:

(2) $$\begin{array}{r} \text{vS}{\text{I}}_{\text{norm}}=\text{Ci}/\text{Cn} \end{array}$$

Another problem with the visual SI measure is that the different age-related trajectories for color vision (declining) and reading speed (stable) mean that color naming RTs in the neutral condition (Cn) may slow with age relative to reading speed (Rn) (Salthouse and Meinz, 1995). The Stroop effect originates from the difference in time course between color naming in the presence vs. absence of a readable distracting color word. If color naming slows while word reading remains unchanged with age, then there will be a greater difference in processing speed between the color naming and reading dimensions, and this puts participants at greater risk of inhibition failure in the incongruent (distractor) condition: that is, if a participant's color naming speed is relatively slow compared to their reading speed, they have to suppress the irrelevant word for longer, and this increases their chances of experiencing an inhibition failure.

Melara and Algom (2003) refer to the discrepancy between access to words and color names as the Dimensional Imbalance (DI) i.e.,

(3) $$\begin{array}{r} \text{DI}=\text{Cn}-\text{Rn} \end{array}$$

Thus, a large DI score indicates a slow color naming speed relative to reading speed. Melara & Algom found DI to be strongly positively correlated with Stroop interference (SI) as measured by (1): larger DI scores (relatively slow color naming speeds) were associated with larger Stroop effects.

If an increased dimensional imbalance indeed contributes to larger SI (inhibitory failure) in older adults, then it needs to be taken into account when calculating inhibition ability. There are two possible ways to do this. The first is to calculate a standardized Ci using the DI score, as follows:

(4) $$\begin{array}{r} \text{vS}{\text{I}}_{\text{standard}}=\text{Ci}/\text{DI} \end{array}$$

This factors out the part of Ci which is determined by DI. As a result, differences in color naming speed relative to reading speed are controlled for, leaving only the portion which represents “true” inhibitory ability.

An alternative approach is to use residuals. For a linear regression modeled as Ci_i_ = α + βDI_i_ + ε_i_, the residuals can be calculated as:

(5) $${\text{vSI}}_{\text{res}}={\text{y}}_{\text{Ci}}-{ŷ}_{\text{Ci}}$$

This method regresses Ci on DI, and then takes the unstandardized residual [i.e., the difference between the observed Ci value (y_Ci_) and the predicted Ci value (ŷ_Ci_)] for each participant. These residuals represent the difference between a participant's observed Ci score relative to what their DI score would predict: a residual near to 0 indicates that the observed Ci score is very similar to what the DI score would predict, suggesting that DI explains almost all of the increase in Ci relative to Cn. A positive residual suggests that the observed Ci score is higher than what could be predicted by DI, indicating “true” inhibitory failure; while a negative residual suggests that the observed Ci is lower than what would be predicted based on DI, and represents “true” inhibitory success. This method thus provides a measure of inhibitory control free from the effects of visual sensory decline. It also accounts for general cognitive slowing since, like (2), it is a relational measure. One issue with this method is that the residual scores depend on the performance of the sample—that is, the predictive relationship between DI and Ci is derived only from the study participants, who may not be representative of the wider population. It would be preferable to independently derive a “gold-standard” relationship between DI and Ci; however, this has not yet been done, and so for the current study we must rely on the data from our sample alone.

#### Calculating auditory stroop scores

The traditional Stroop interference measure (SI) for the auditory Stroop is calculated analogously to the visual Stroop:

(6) $$\begin{array}{r} \text{aS}{\text{I}}_{\text{raw}}=\text{aRTi}-\text{aRTn} \end{array}$$

As explained above, the issue of generalized slowing makes the traditional Stroop (SI) measure problematic: if aRTi and aRTn increase by the same factor, SI will also increase; this means that a larger SI may reflect slowing rather than paucity of inhibition. Normalized SI was proposed as one means of addressing the issue of generalized slowing, and can be calculated for the auditory Stroop as follows:

(7) $$\begin{array}{r} \text{aS}{\text{I}}_{\text{norm}}=\text{aRTi}/\text{aRTn} \end{array}$$

As discussed in the Introduction, using average measures across all trials of a Stroop task may not be the most efficient way of quantifying inhibition and its failure. We know that inhibition takes time to build up, and that its effects may therefore be strongest for each participant's slowest RTs for incongruent trials (Ridderinkhof, 2002; Ridderinkhof et al., 2004; Roelofs et al., 2011). During these trials the distractor has the greatest chance to interfere, but inhibition also has the greatest potential to be deployed by those who can successfully do so; thus individual differences in inhibitory abilities will be most in evidence, since the disparity between those able to successfully deploy inhibition and those less able to do so will be largest during these trials (Roelofs et al., 2011). To assess this, slow and fast trials must be analyzed separately. This type of differential analysis of single trials is usually done using delta plots and delta scores.

Delta scores are calculated using neutral (aRTn) and incongruent (aRTi) conditions. For each participant and each condition, the trials are sorted by RT, and then split into equally-sized quintiles. The average RT is calculated for each quintile in each condition. Mean RT per quintile is the averaged RT across aRTn and aRTi for a given quintile. Delta RT per quintile is calculated as mean aRTi minus mean aRTn for a given quintile. When averaged over all participants the grand mean RT and grand delta RT can be obtained for each quintile. It is worth noting that, since delta RT per quintile is obtained by calculating aRTi—aRTn for that quintile, it is conceptually no different to using the traditional (aSI_raw_) measure (see equation (6) above). It is the same calculation, but performed using only a subset of trials.

Delta plots show grand mean RTs plotted against grand delta RTs for the five RT quintiles (Q1-Q5). Since the delta RT measure compares conditions with and without distractors, and interference from distractors increases over time, the plots typically show an overall increase in delta RTs as mean RTs increase. Individual differences in the build-up of inhibition are expressed in a delta plot by differences in this relationship between mean and delta RTs (Ridderinkhof et al., 2004). Those who are not successfully inhibiting show a monotonic increase in delta RT as mean RT increases. In contrast, those who are successfully engaging inhibition initially show a monotonic increase in delta RT, but for the slowest trials the relationship between delta RT and mean RT will become less steep, flatten out or even become negative. Delta plots allow us to focus on those trials that both allow and require the most inhibition for successful performance, thereby maximizing the chance of seeing individual differences in inhibitory ability.

### Speech-in-noise tasks

The SiN tasks varied in both semantic context and lexical difficulty. Semantic context was varied as part of the sentence task, where target words were the final words of low- (LP) and high-predictability (HP) sentences. Stimuli were 112 sentence pairs from a recently developed sentence pairs test (Heinrich et al., 2014). This test, based on the SPIN-R test (Bilger et al., 1984), comprises sentence pairs with identical sentence-final monosyllabic words, which are more or less predictable from the preceding context (e.g., “We'll never get there at this rate” vs. “He's always had it at this rate”). High and low predictability (HP/LP) sentence pairs were matched for duration, stress pattern, and semantic complexity. Sentences were recorded using a male Standard British English speaker. Only the HP or LP version of a sentence was heard by a single participant.

Lexical difficulty was assessed in the word task, where target stimuli were 200 isolated words whose lexical difficulty was varied in terms of word frequency (WF) and neighborhood density (ND). The set of words comprised the 112 final words from the sentence task and an additional 88 monosyllables. WF was measured using the BNC corpus (http://www.natcorp.ox.ac.uk/), filtered for nouns (exact form). This corpus was chosen because it both uses British English and also allows particular parts of speech to be isolated: in this case, the measure of interest was the frequency of the target words as nouns, since the sentence contexts led listeners to anticipate a noun target, and as the exact form heard in the sentence, not with potential pluralizations or any other alterations. This limitation was mirrored in the scoring of the SiN task, where only the exact form of a target was scored as correct. ND was determined using N-Watch (Davis, 2005). This tool uses the Celex database to create neighborhood measures using a letter-substitution algorithm, but cross-checks the measures with word frequency to ensure that extremely rare words are not included. This stops over-estimation of ND with respect to most people's vocabulary. It also uses British English. Based on these measures, the 200 words were divided into 4 groups, with WF and ND ranges as shown in Table 1.

**Table 1:** **Lexical information for word stimuli**.

		**Low WF Low ND**	**Low WF High ND**	**High WF Low ND**	**High WF High ND**
WF	Max	9,879	8,958	41,358	62,803
	Min	106	117	10,152	10,029
ND	Max	18	38	18	35
	Min	2	19	2	19

All 200 words were re-recorded using a different male Standard British English speaker.

All SiN stimuli were presented in speech-modulated noise (SMN). The SMN was created by using an inverse FFT to generate a noise signal with the same long-term average spectrum as the target speech. This noise signal was then modulated in level by dot multiplying it with the absolute value of the smoothed Hilbert transform of the target speech (smoothing was accomplished by convolving the speech envelope with a 46 ms vector of ones). Finally the SMN was scaled to match the RMS level of the target speech. This made the speech signal unintelligible while keeping the long-term average spectrum, level, and temporal envelope of the original signal intact. SiN stimuli were presented in two SNRs to create a more or less adverse listening condition (words at +1 and −2 dB; sentences at −4 and −7 dB). SNR levels were chosen to vary the overall difficulty of the task between 20 and 80% accuracy. Each of the 112 sentence-final words was only heard once by each participant, either in the context of an HP or an LP sentence, and half the sentences of each type were heard with high or low SNR. Each of the 200 words was heard only once, with either high or low SNR, and there were equal numbers of words in each combination of word frequency and neighborhood density categories. After hearing each sentence or word participants repeated as much as they could. Testing was self-paced, and responses were recorded for offline scoring.

### Procedure

Testing was carried out in a double-wall sound-attenuating booth (Industrial Acoustics Company (IAC), Winchester, UK) using Sennheiser HD280 headphones. All testing was in the left ear only. The SiN and Stroop tasks formed part of a larger battery of tests, which were administered over the course of two sessions around a week apart. The two SiN tasks (words and sentences) were always tested in different sessions; the two Stroop tasks (auditory and visual) were tested in different sessions wherever possible, which was the majority of cases. The order of SiN tasks was counterbalanced across participants. There was no systematic pairing of SiN and Stroop tasks within sessions.

### Modeling

In all cases, the outcome measure was speech intelligibility as measured in RAUs (Studebaker, 1985). A number of stimulus-based variables were coded as categorical predictors: semantic predictability (LP/HP) of sentence-final words; word frequency (high/low) and neighborhood density (high/low) of isolated words; speech type (sentences/words) of words and sentences; SNR (high/low). In addition, the following listener variables were coded as continuous predictors: Stroop score (on either the auditory or visual Stroop tasks, using a specified scoring system), and PTA. The PTA variable was calculated by averaging the obtained thresholds at all tested frequencies for each participant, and then centering these values.

The relationship between predictor and outcome variables was assessed in a series of linear mixed models (LMMs) using ML estimation, with predictor variables as fixed effects and Type 3 SS. All models included participants as random effects.

A backwards stepwise procedure was used to determine the final set of predictors for each model.^1^ This procedure was implemented through manual checking and effect removal. All analyses were performed in IBM SPSS Statistics 21.

## Results

### Mean results for speech-in-noise (SiN) perception

Mean intelligibility values for all SiN conditions are given in Table 2.

**Table 2:** **Mean scores and standard errors in the 6 different SiN conditions**.

**Sentences**	**Semantic predictability**		**Easy SNR (–4 dB)**		**Hard SNR (–7 dB)**	
			**Mean**	**SE**	**Mean**	**SE**
	HP		0.88	0.015	0.73	0.024
	LP		0.57	0.018	0.41	0.018
**Words**	**Word frequency**	**Neighborhood density**	**Easy SNR (+1 dB)**		**Hard SNR (–2 dB)**	
	High WF	High ND	0.71	0.017	0.58	0.018
	High WF	Low ND	0.82	0.016	0.76	0.017
	Low WF	High ND	0.72	0.018	0.64	0.021
	Low WF	Low ND	0.67	0.015	0.60	0.020

Repeated-measures ANOVAs were conducted to investigate group differences in word and sentence intelligibility due to stimulus-based predictor variables. For intelligibility of sentence-final words, a semantic predictability (LP/HP) x SNR (low/high) within-subjects ANOVA showed significant main effects of both predictability [*F*_(1, 49)_ = 571.72; *MSE* = 91.67, *p* < 0.001, η^2^ = 0.921; HP > LP] and SNR [*F*_(1, 49)_ = 168.54; *MSE* = 76.81, *p* < 0.001, η^2^ = 0.775; easy > hard], but no predictability × SNR interaction. For intelligibility of isolated words, a word frequency (low/high) × neighborhood density (low/high) × SNR (low/high) within-subject ANOVA showed significant main effects of word frequency (WF) [*F*_(1, 49)_ = 111.67; *MSE* = 37.37, *p* < 0.001, η^2^ = 0.695; high > low], neighborhood density (ND) [*F*_(1, 49)_ = 33.89; *MSE* = 70.11, *p* < 0.001, η^2^ = 0.409; low > high] and SNR [*F*_(1, 49)_ = 120.69; *MSE* = 66.54, *p* < 0.001, η^2^ = 0.711; easy > hard]; additionally, a significant WF × ND interaction [*F*_(1, 49)_ = 180.40; *MSE* = 54.53, *p* < 0.001, η^2^ = 0.786] indicated that words with both a high word frequency and a low neighborhood density were more intelligible than words in the other three conditions (Bonferroni-corrected at *p* = 0.05).

### Visual stroop

The mean for Cn was 31.66 s (*SD* = 5.41 s); the mean for Ci was 47.13 s (*SD* = 8.14 s); and in all cases the difference between them was positive (i.e., Ci > Cn). The mean difference between RTs in the two conditions for the current dataset was 15.5 s (*SD* = 4.49 s) overall, which represents a mean of 0.32 s (*SD* = 0.09 s) per item (word). The vSI_norm_ measure (Equation 2 above) gives a mean score of 1.49 (*SD* = 0.14).

#### The relationship between visual stroop scores and speech-in-noise (SiN) perception

This section examines the predictive value of visual Stroop interference for SiN perception in high and low predictability sentences and for single words varying in word frequency and neighborhood density. Predictive power for SiN perception was investigated for two measures of visual Stroop interference: vSI_raw_, the traditional measure of Stroop interference unadjusted for sensory decline, and vSI_res_, the new measure of Stroop interference that takes general age-related slowing as well as sensory decline into account. The predictive relationship between each of the visual Stroop scores and performance on the sentence task, the word task and the sentence and word tasks combined are presented in Tables 3–5 respectively. The analyses combining the scores from the sentence and word tasks (Table 5) were included in order to directly compare the predictive effect of Stroop scores across target stimuli of different linguistic complexity. In a second step, PTA was added to each set of analyses in order to examine how it modified the predictive effect of the Stroop scores.

**Table 3:** **Summary of LMMs assessing relationship of visual Stroop scores to sentence perception**.

	**AIC value**	**ME**	**Interaction(s) involving stroop**	**Description**
Scoring method: vSI_raw_	**Listener-based predictors:** Stroop			
	1426.747	N	N	N/A
	**Listener-based predictors:** Stroop, PTA			
	1394.693	N	N	N/A
Scoring method: vSI_res_	**Listener-based predictors:** Stroop			
	1429.328	N	(1) vSI_res_*Pred*SNR	(1) At the high (easy) SNR, the slope predicting SiN performance from Stroop interference is positive for HP sentences and negative for LP. At the low (hard) SNR, the slope is negative for HP and positive for LP
	**Listener-based predictors:** Stroop, PTA			
	1396.551	N	(1) vSI_res_*Pred*SNR	(1) As above

*Stimulus-based predictors: semantic predictability (high/low), SNR (high/low)*.

**Table 4:** **Summary of LMMs assessing relationship of visual Stroop scores to word perception**.

	**AIC value**	**ME**	**Interaction(s) involving stroop**	**Description**
Scoring method: vSI_raw_	**Listener-based predictors**: Stroop			
	2708.973	N	(1) vSI_raw_*ND	(1) The slope predicting SiN performance from Stroop interference is negative overall, and most strongly so for words with low neighborhood density (ND)
	**Listener-based predictors:** Stroop, PTA			
	2695.725	N	(1) vSI_raw_*ND (2) vSI_raw_*SNR*ND*PTA	(1) The slope predicting SiN performance from Stroop interference is negative overall, and most strongly so for words with low neighborhood density (ND) (2) For those with poor PTA, the slope predicting SiN performance from Stroop interference is negative and stronger for low ND words For those with good PTA, the slope is positive for high ND words and negative for low ND words at the easier SNR, and approaches zero for both ND categories at the harder SNR
Scoring method: vSI_res_	**Listener-based predictors:** Stroop			
	2712.168	N	N	N/A
	**Listener-based predictors:** Stroop, PTA			
	2691.369	N	(1) vSI_res_*ND (2) vSI_res_*ND*PTA	(1) The slope predicting SiN performance from Stroop interference is negative overall, and most strongly so for words with low neighborhood density (ND) (2) For those with good PTA, the slope predicting SiN performance from Stroop interference is negative for both ND categories For those with poor PTA, the slope is more strongly negative for low ND words and approaches zero for high ND words

*Stimulus-based predictors: word frequency (high/low), neighborhood density (high/low), SNR (high/low)*.

**Table 5:** **Summary of LMMs assessing relationship of visual Stroop scores to all SiN perception (combined dataset)**.

	**AIC value**	**ME**	**Interaction(s) involving stroop**	**Description**
Scoring method: vSI_raw_	**Listener-based predictors:** Stroop			
	1266.480	N	(1) vSI_raw_*Type	(1) The slope predicting SiN performance from Stroop interference is negative for words and mildly positive for sentences
	**Listener-based predictors:** Stroop, PTA			
	1236.257	N	(1) vSI_raw_ *Type	(1) As above
Scoring method: vSI_res_	**Listener-based predictors:** Stroop			
	1270.403	N	N	N/A
	**Listener-based predictors:** Stroop, PTA			
	1239.501	N	N	N/A

*Stimulus-based predictors: type (sentences/words), SNR (high/low)*.

Tables 3–5 indicate, for each combination of model type and dataset, (a) whether a predictive effect of the Stroop measure on SiN performance was present, and what the nature of the effect was; and (b) what, if any, significant interactions between the Stroop measure and stimulus-based variables or PTA were present. The effects are described as rate of change where a positive slope indicates an average increase in SiN performance with every additional increase in Stroop interference, while a negative slope indicates an average decrease in SiN performance with every additional increase in Stroop interference. Based on our hypotheses, we expect negative slopes. While PTA was always entered as a continuous predictor, we use a categorical median split when reporting and discussing its effects, because it allows for clearer descriptions, particularly of complex interactions. The tables do not list significant interactions if they do not involve the Stroop measure. The *AIC* value is included for each model as an indication of goodness-of-fit, with lower *AIC* values corresponding to a better fit.

The models reveal a complex pattern of results with the direction of the relationship between the vSI measures and SiN performance, as well as the strength of the relationship, depending on the scoring method and characteristics of the stimulus and the listener. However, in all cases, the inclusion of PTA in the model enhanced model fit (i.e., produced a lower *AIC* value).

We will now examine, for each dataset in turn, how the nature of the relationship between Stroop scores and SiN performance was modulated by stimulus-based variables and PTA for each Stroop scoring method.

##### Sentence perception

*Traditional (vSI_raw_) measure*. There was no predictive effect of the Stroop measure overall, and stimulus-based predictors did not modulate the predictive effect of Stroop interference. There was also no modulating effect of PTA.

*Adjusted (vSI_res_) measure*. While there was no predictive main effect of Stroop interference, an interaction of vSI_res_∗Pred∗SNR indicates that the predicted negative relationship between Stroop scores and sentence perception was seen for the high predictability (HP) sentences in the harder SNR, and for the low predictability (LP) sentences in the easier SNR, but not for the HP sentences in the easier SNR or the LP sentences in the harder SNR. There was no modulating effect of PTA.

##### Word perception

*Traditional (vSI_raw_) measure*. While there was no predictive main effect of Stroop interference, an interaction with neighborhood density (ND) indicates that the observed relationship between vSI_raw_ and word perception was more negative for words with less dense neighborhoods. This interaction was modulated by SNR and PTA in an interaction of vSI_raw_∗SNR∗ND∗PTA, indicating that the relationship between Stroop scores and SiN perception changed in different ways across ND and SNR conditions for listeners with better and worse hearing. Specifically, the relationship was negative for those with poor PTA, but was more mixed for those with good PTA, being positive for high ND words in the easier SNR and approaching zero for both ND conditions in the harder SNR.

*Adjusted (vSI_res_) measure*. There was no main effect of Stroop interference and no modulating effects of stimulus-based variables on their own. Once PTA was added to the model, an interaction of vSI_res_∗ND emerged, indicating that the predictive effect of Stroop scores was strongest for low ND words. This interaction was further modulated by PTA, indicating that the relationship between Stroop scores and SiN perception changed in different ways for the two ND conditions when examining listeners with better and worse hearing. Specifically, for those with worse hearing the Stroop/SiN relationship was more negative for low ND words but less negative for high ND words when compared to those with better hearing.

##### Speech (combined dataset)

*Traditional (vSI_raw_) measure*. There was no predictive main effect of Stroop interference. An interaction with Type indicates that the predictive effect of Stroop scores for SiN perception differed in direction between sentences and words, being negative for the word task and positive for the sentence task. PTA did not modulate the found relationships.

*Adjusted (vSI_res_) measure*. There was no main effect of Stroop interference and no modulating effects of stimulus-based variables or PTA.

In summary, the predictive effect of visual Stroop scores for SiN perception is similar in some respects across all three analyses and regardless of the scoring method. Both scoring systems reveal some specific influences of lexical factors [sentence predictability (Table 3) and word neighborhood density (Table 4)], and neither system shows a large effect of PTA.

### Auditory stroop (all trials)

The auditory Stroop task resulted in three measures for each participant: average RT for neutral trials (aRTn), congruent trials (aRTc) and incongruent trials (aRTi). Initial inspection of the data revealed that not all four speakers produced Stroop interference effects for every participant. We therefore analyzed for each participant the responses to the female and male speaker who produced, for that participant, the largest overall traditional Stroop interference (aRTi—aRTn). Speakers M1 and M2 were chosen 13 and 35 times respectively, speakers F1 and F2 25 and 23 times respectively. Following Green and Barber (1981), only correct trials from the aRTi and aRTn conditions were included in any analysis.

Congruent trials are usually included in Auditory Stroop tasks, and previous studies (Green and Barber, 1981; Jerger et al., 1988) have found a facilitation effect (i.e., faster responses to congruent than neutral trials), although this is not always the case (Sommers and Danielson, 1999). Using a 1-way repeated-measures ANOVA (Greenhouse-Geisser corrected for violations of sphericity) with aRTn, aRTc and aRTi as within-subject levels of condition, we found a main effect of condition [*F*_(2, 79)_ = 53.40; *MSE* = 0.005, *p* < 0.001, η^2^ = 0.532]. *Post-hoc* testing showed an interference effect but no facilitation effect [aRTi > aRTc, aRTi > aRTn, aRTc = aRTn (Bonferroni-corrected at *p* = 0.05)].

The mean aRTi (per item) was 1.33 s (*SD* = 0.23 s), the mean aRTn was 1.20 s (*SD* = 0.21 s), and aRTi was higher than aRTnfor all but 3 listeners. The mean difference between RTs in the two conditions for the current dataset was 0.13 s (*SD* = 0.09 s) per item (word). This difference is smaller than for the visual Stroop. The aSI_norm_ measure (Equation 7 above) gives a mean score of 1.11 (*SD* = 0.08).

#### The relationship between auditory stroop scores and speech-in-noise (SiN) perception

This section examines the predictive value of auditory Stroop interference for SiN perception in high and low predictability sentences, and for single words varying in word frequency and neighborhood density. As before, performance in these conditions was predicted by one of two auditory Stroop interference measures: aSI_raw_, the traditional measure for Stroop interference, or aSI_norm_, a measure of Stroop interference that takes generalized slowing into account. The relationship between each Stroop measure and SiN perception, as characterized by a series of LMMs, is summarized in Tables 6–8. In all cases, the first part of the table presents the results when Stroop interference and stimulus-based variables are the only predictors of SiN performance. The second part of each table presents the results when PTA is considered in addition to Stroop interference and stimulus-based variables.

**Table 6:** **Summary of LMMs assessing relationship of auditory Stroop scores to sentence perception**.

	**AIC value**	**ME**	**Interaction(s) involving stroop**	**Description**
Scoring method: aSI_raw_	**Listener-based predictors:** Stroop			
	1459.850	N	N	N/A
	**Listener-based predictors:** Stroop, PTA			
	1428.302	N	N	N/A
Scoring method: aSI_norm_	**Listener-based predictors:** Stroop			
	1456.132	Y	N	N/A
	**Listener-based predictors:** Stroop, PTA			
	1427.957	N	(1) aSI_norm_∗Pred∗SNR∗PTA	(1) For those with good PTA, the slope predicting SiN performance from Stroop interference is positive for HP sentences at the easier SNR and LP sentences at the harder SNR, and approaches zero elsewhere For those with poor PTA, the slope is positive for HP sentences at the harder SNR and LP sentences for the easier SNR, and approaches zero elsewhere

*Stimulus-based predictors: predictability (high/low), SNR (high/low)*.

**Table 7:** **Summary of LMMs assessing relationship of auditory Stroop scores to word perception**.

	**AIC value**	**ME**	**Interaction(s) involving stroop**	**Description**
Scoring method: aSI_raw_	**Listener-based predictors:** Stroop			
	2776.946	N	(1) aSI_raw_∗SNR	(1) The slope predicting SiN performance from Stroop interference is positive overall, and more strongly so at the harder SNR
	**Listener-based predictors: Stroop, PTA**			
	2759.515	N	(1) aSI_raw_∗SNR	(1) As above
Scoring method: aSI_norm_	**Listener-based predictors: Stroop**			
	2771.321	Y	(1) aSI_norm_∗SNR	(1) The slope predicting SiN performance from Stroop interference is positive in both conditions, and more strongly so at the harder SNR
	**Listener-based predictors: Stroop, PTA**			
	2755.034	N	(1) aSI_norm_∗SNR	(1) As above

*Stimulus-based predictors: word frequency (high/low), neighborhood density (high/low), SNR (high/low)*.

**Table 8:** **Summary of LMMs assessing relationship of auditory Stroop scores to all SiN perception (combined dataset)**.

	**AIC value**	**ME**	**Interaction(s) involving stroop**	**Description**
Scoring method: aSI_raw_	**Listener-based predictors:** Stroop			
	1289.565	N	(1) aSI_raw_∗SNR	(1) The slope predicting SiN performance from Stroop interference is positive overall, and more strongly so for the harder SNR
	**Listener-based predictors:** Stroop, PTA			
	1260.049	N	(1) aSI_raw_∗SNR	(1) As above
Scoring method: aSI_norm_	**Listener-based predictors:** Stroop			
	1285.224	Y	(1) aSI_norm_∗SNR	(1) The slope predicting SiN performance from Stroop interference is positive overall, and more strongly so for the harder SNR
	**Listener-based predictors:** Stroop, PTA			
	1256.700	Y	(1) aSI_norm_∗SNR	(1) As above

*Stimulus-based predictors: type (sentences/words), SNR (high/low)*.

For both auditory Stroop scoring systems, the overall relationship between Stroop scores and SiN perception is mostly positive. This is truer for the normalized (aSI_norm_) scores than the traditional (aSI_raw_) scores, since Stroop scores never reach significance as a main effect when using the aSI_raw_ scoring method, but are significant across all datasets when using the aSI_norm_ measure without PTA. As before, including PTA improved the fit of the model in all cases.

We will now examine, for each dataset in turn, how the nature of the relationship between Stroop scores and SiN performance was modulated by stimulus-based variables and PTA for each Stroop scoring method.

##### Sentence perception

*Traditional (aSI_raw_) measure*. There was no main effect of Stroop interference and no modulating effects of stimulus-based variables or PTA.

*Adjusted (aSI_norm_) measure*. There was a positive predictive main effect of Stroop scores but no modulating effects of stimulus-based variables on their own. When PTA was added as an additional predictor an interaction of aSI_norm_∗Pred∗SNR∗PTA emerged, which indicates that the predictive strength, but not the direction, of Stroop interference for speech perception in a particular condition depended on a person's hearing sensitivity.

##### Word perception

*Traditional (aSI_raw_) measure*. While there was no predictive main effect, an interaction of aSI_raw_∗SNR indicates that the positive predictive effect of Stroop scores on SiN performance was stronger at the harder SNR. There was no modulating effect of PTA.

*Adjusted (aSI_norm_) measure*. As for aSI_raw_ above, but with a positive predictive main effect of Stroop scores before the addition of PTA.

##### Speech (combined dataset)

*Traditional (aSI_raw_) measure*. Again, there was no predictive main effect of Stroop, but an interaction with SNR indicates a stronger positive predictive effect at the harder SNR. There was no modulating effect of PTA

*Adjusted (aSI_norm_) measure*. As for aSI_raw_ above, but with a positive predictive main effect of Stroop scores.

In summary, the predictive relationship between auditory Stroop scores and SiN perception is in some ways similar for auditory Stroop scores calculated using the traditional method (aSI_raw_) and the normalization method (aSI_norm_). For both scoring methods, the Stroop/SiN relationship is positive overall and stronger at the more challenging SNR. However, there are also differences. In particular, the traditional Stroop scores (aSI_raw_) did not appear to predict performance on the sentence task, unlike the aSI_norm_ scores (Table 6). However, there was no interaction of speech type with Stroop scores from either scoring method when examining the combined dataset (Table 8), so this apparent disparity between the two scoring systems should be treated with caution.

### Auditory stroop (slow vs. fast trials)

As discussed in the Materials and Methods section, delta scores can be used to examine Stroop interference (SI) in different subsets of trials from a Stroop task. Conceptually, these delta scores are the same as the traditional (aSI_raw_) measure, but calculated using only those trials which fall in a given section of a participant's RT distribution. We are interested in assessing SI derived from the slowest quintile (Q5) and fastest quintile (Q1) of each participant's trials. The slowest trials are used because individual differences in performance on inhibition tasks have been shown to be greatest in this quintile (Ridderinkhof, 2002; Ridderinkhof et al., 2004), thus giving us better statistical power to observe links with SiN perception. This larger variation in individual differences is hypothesized to be due to slow trials better revealing individual differences in inhibition (Ridderinkhof et al., 2004; Roelofs et al., 2011). For this reason, we hypothesize that delta scores from Q5 will correlate more strongly with SiN perception than scores from Q1: that is, if SiN perception is determined, at least in part, by inhibitory ability, then SiN scores should correlate more strongly with measures which better reveal differences in inhibitory ability.

**Figure 2:**
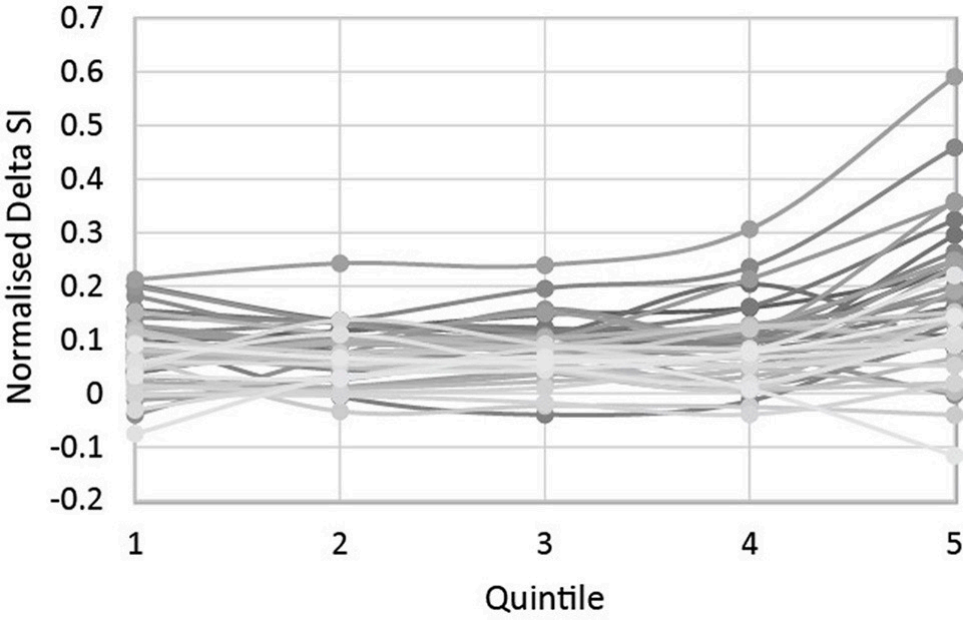
**Each individual's aSI_ndelta_ scores across the five quintiles**.

Because participants varied widely in overall RTs, we divided each delta RT by its relevant mean RT to get a normalized delta score, called hereafter aSI_ndelta_. These scores are plotted in Figure 2.

A repeated-measures 1-way ANOVA with quintiles as within-subject effects (Greenhouse-Geisser corrected for violations of sphericity) showed a main effect of quintile [*F*_(2, 84)_ = 18.69, *MSE* = 0.007; *p* < 0.001, η^2^ = 0.284], and subsequent pairwise comparisons (Bonferroni corrected at *p* = 0.05) revealed that Q5 had significantly higher normalized delta scores compared to all other quintiles, which were not significantly different from each other. However, as Figure 2 shows, Q5 produced not only the largest delta scores (largest Stroop effects) on average, but also the largest variation in scores: the standard deviation of scores in Q5 is 0.12 s, compared to a range of 0.05–0.07 for Q1-4. This is in concordance with Ridderinkhof et al. (2004), and also suggests that Q5 is most likely to reveal differential associations between the auditory Stroop measure and SiN perception. If Ridderinkhof and colleagues are correct that there is not enough time for inhibition to become sufficiently strong and/or be successfully deployed during participants' fastest responses, then Q1 should not only show smaller Stroop effects on average and a limited variation in scores, as demonstrated above, but should also have only limited predictive value for performance on the SiN perception tasks.

#### The relationship between auditory stroop delta scores and speech-in-noise (SiN) perception

This section examines the predictive value of the two auditory Stroop delta score measures for SiN perception in the six SiN conditions. Two auditory Stroop interference measures were used: aSI_ndeltaQ5_ as a measure of interference derived from the slowest trials; and aSI_ndeltaQ1_ as a measure of interference derived from the fastest trials. The relationship between each of these measures and SiN perception, as characterized by a series of LMMs, is summarized in Tables 9–11.

**Table 9:** **Summary of LMMs assessing relationship of auditory Stroop delta scores to sentence perception**.

	**AIC value**	**ME**	**Interaction(s) involving stroop**	**Description**
Scoring method: aSI_ndeltaQ5_	**Listener-based predictors:** Stroop			
	1493.843	N	(1) aSI_ndeltaQ5_∗Pred∗SNR	(1) The slope predicting SiN perception from Stroop interference is positive for LP sentences at the easier SNR and HP sentences at the harder SNR, and approaches zero elsewhere
	**Listener-based predictors:** Stroop, PTA			
	1457.746	N	(1) aSI_ndeltaQ5_∗Pred∗SNR	(1) As above
Scoring method: aSI_ndeltaQ1_	**Listener-based predictors:** Stroop			
	1491.747	N	N	N/A
	**Listener-based predictors:** Stroop, PTA			
	1458.472	N	N	N/A

*Stimulus-based predictors: predictability (high/low), SNR (high/low)*.

**Table 10:** **Summary of LMMs assessing relationship of auditory Stroop delta scores to word perception**.

	**AIC value**	**ME**	**Interaction(s) involving stroop**	**Description**
Scoring method: aSI_ndeltaQ5_	**Listener-based predictors:** Stroop			
	2827.234	Y	(1) aSI_ndeltaQ5_∗SNR	(1) The slope predicting SiN perception from Stroop interference is positive overall, and more strongly so at the harder SNR
	**Listener-based predictors:** Stroop, PTA			
	2807.669	Y	(1) aSI_ndeltaQ5_∗SNR	(1) As above
Scoring method: aSI_ndeltaQ1_	**Listener-based predictors:** Stroop			
	2833.745	N	N	N/A
	**Listener-based predictors:** Stroop, PTA			
	2817.638	N	N	N/A

*Stimulus-based predictors: word frequency (high/low), neighborhood density (high/low), SNR (high/low)*.

**Table 11:** **Summary of LMMs assessing relationship of auditory Stroop delta scores to all SiN perception (combined dataset)**.

	**AIC value**	**ME**	**Interaction(s) involving stroop**	**Description**
Scoring method: aSI_ndeltaQ5_	**Listener-based predictors:** Stroop			
	1321.151	N	(1) aSI_ndeltaQ5_∗Type (2) aSI_ndeltaQ5_∗SNR	(1) The slope predicting SiN perception from Stroop interference is positive overall, and more strongly so for words (2) The slope predicting SiN perception from Stroop interference is positive overall, and more strongly so for the harder SNR
	**Listener-based predictors:** Stroop, PTA			
	1282.466	N	(1) aSI_ndeltaQ5_∗SNR (2) aSI_ndeltaQ5_∗Type∗PTA	(1) As above (2) For those with good PTA, the slope predicting SiN perception from Stroop interference is positive and stronger for sentences For those with poor PTA, the slope is positive and stronger for words
Scoring method: aSI_ndeltaQ1_	**Listener-based predictors:** Stroop			
	1325.809	N	N	N/A
	**Listener-based predictors:** Stroop, PTA			
	1294.172	N	(1) aSI_ndeltaQ1_∗Type∗PTA	(1) For those with good PTA, the slope predicting SiN perception from Stroop interference is negative and stronger for words For those with poor PTA, the slope is negative and stronger for sentences

*Stimulus-based predictors: type (sentences/words), SNR (high/low)*.

We will now examine, for each dataset in turn, how the nature of the relationship between Stroop scores and SiN performance was modulated by stimulus-based variables and PTA for each Stroop scoring method.

##### Sentence perception

*Slowest (aSI_ndeltaQ5_) trials*. While there was no predictive main effect of the Stroop measure, an interaction of aSI_ndeltaQ5_∗Pred∗SNR indicates that the positive slope predicting SiN performance from Stroop interference was steeper for high predictability (HP) sentences in the more challenging SNR, and for low predictability (LP) sentences in the easier SNR. There was no additional modulating effect of PTA.

*Fastest (aSI_ndeltaQ1_) trials*. There was no main effect of Stroop interference and no modulating effects of stimulus-based variables or PTA.

##### Word perception

*Slowest (aSI_ndeltaQ5_) trials*. In addition to a positive predictive main effect of Stroop scores, an interaction of aSI_ndeltaQ5_∗SNR indicates that the positive slope predicting SiN performance from Stroop interference was steeper at the harder SNR. This interaction was not modulated by PTA.

*Fastest (aSI_ndeltaQ1_) trials*. There was no main effect of Stroop interference and no modulating effects of stimulus-based variables or PTA.

##### Speech (combined dataset)

*Slowest (aSI_ndeltaQ5_) trials*. There was no predictive main effect of Stroop. An interaction with Type indicates that there was a stronger positive predictive effect of Stroop scores for SiN perception in the word than the sentence task. An interaction with SNR indicates that the positive predictive effect of Stroop scores on SiN performance was stronger at the harder SNR. The interaction with Type was modulated by PTA, indicating that the Stroop/SiN relationship varied in strength across SiN type and levels of hearing loss, but remained positive throughout.

*Fastest (aSI_ndeltaQ1_) trials*. There was no main effect of Stroop interference and no modulating effects of stimulus-based variables on their own. Once PTA was added, an interaction of aSI_ndeltaQ1_∗Type∗PTA emerged, indicating that the relationship between Stroop scores and SiN perception varied in strength across SiN type and levels of hearing sensitivity, but remained negative overall.

In summary, the relationship between auditory Stroop scores and SiN perception varies considerably depending on whether the auditory Stroop scores are calculated using either only the slowest responses (aSI_ndeltaQ5_) or only the fastest responses (aSI_ndeltaQ1_). First, for aSI_ndeltaQ5_, the Stroop/SiN relationship is positive overall, stronger for words than sentences for those with poor hearing, and stronger at the more challenging SNR. This stands in contrast to the aSI_ndeltaQ1_ scores, for which the Stroop/SiN relationship is negative overall, stronger for sentences than words for those with poor hearing, and unaffected by SNR. Second, the aSI_ndeltaQ1_ scores have no predictive value for performance on the sentence task, whereas the aSI_ndeltaQ5_ are significantly related to sentence perception. Finally, it is worth noting that the aSI_ndeltaQ1_ scoring method reveals a mixture of positive and negative Stroop/SiN relationships. However, for aSI_ndeltaQ5_—the scoring method which uses only the very slowest trials—the relationship between Stroop scores and SiN perception is almost always positive.

### Intercorrelations of stroop scoring systems

Table 12 shows the intercorrelations of all six Stroop scoring systems used in the current study. The scores for the two visual Stroop scoring methods, vSI_raw_ and vSI_res_, are highly positively correlated. The scores for the two auditory Stroop scoring methods which use data from all trials, aSI_raw_ and aSI_norm_, are also highly correlated. The auditory Stroop scores which use data from all trials are also highly correlated with the auditory Stroop score derived from the slowest trials (aSI_ndeltaQ5_), and moderately correlated with the auditory Stroop scores derived from the fastest trials (aSI_ndeltaQ1_). However, the scores from the slow and fast trials (aSI_ndeltaQ5_ and aSI_ndeltaQ1_) are not correlated with each other. There are no significant correlations between the scores from either of the visual Stroop scoring systems and any of the scores from the auditory Stroop scoring systems.

**Table 12:** **Intercorrelations of all Stroop scoring systems (visual and auditory)**.

	**vSI_raw_**	**vSI_res_**	**aSI_raw_**	**aSI_norm_**	**aSI_ndeltaQ5_**	**aSI_ndeltaQ1_**
vSI_raw_	−					
vSI_res_	0.763^**^	−				
aSI_raw_	−0.013	0.050	−			
aSI_norm_	−0.009	0.008	0.953^**^	−		
aSI_ndeltaQ5_	−0.265	−0.213	0.815^**^	0.850^**^	−	
aSI_ndeltaQ1_	0.208	0.117	0.384^**^	0.406^**^	0.202	−

** *p < 0.01*.

## Discussion

Inhibition is a key cognitive ability, and has been suggested to be important for speech-in-noise perception. However, existing attempts to connect inhibitory abilities to performance on speech-in-noise tasks may have been complicated by methodological issues regarding the use of Stroop tasks. One widely-used method for measuring inhibition is the color-word Stroop task (Stroop, 1935), which uses visual stimuli and exploits the difference in processing time between reading and color naming. More recently, auditory Stroop tasks have been developed (Green and Barber, 1981; Morgan and Brandt, 1989) that are designed to measure auditory inhibitory abilities. However, the relationship between these two types of Stroop task, and the question of whether or not they assess the same underlying ability, is not clear. Another issue concerning all Stroop tasks is the question of which scoring system is the most appropriate for estimating inhibitory ability independent of sensory contributions. This question is particularly pertinent to research involving older adults, where it is important not to misattribute sensory changes to changes in cognition. Here we set out to investigate both of these questions—that is, whether auditory and visual Stroop tasks assess similar aspects of an underlying concept, and how the use of different scoring systems that either do or do not take sensory changes into account affects the results. In all cases, the outcome of interest was the way in which a particular Stroop task analyzed using a particular scoring method related to and predicted performance on a set of speech-in-noise tasks.

We used two Stroop tasks, a visual and an auditory. For the visual Stroop task we explored two scoring methods: the traditional Stroop interference measure (vSI_raw_), and a residuals-based measure designed to account for both generalized slowing and declines in color vision (vSI_res_). For the auditory Stroop data, we explored four scoring methods: the traditional Stroop interference measure (aSI_raw_), a normalized version of the traditional measure designed to account for generalized slowing (aSI_norm_), a normalized measure of interference for each participant's slowest trials (aSI_ndeltaQ5_) and a normalized measure of interference for each participant's fastest trials (aSI_ndeltaQ1_).

The speech tasks were selected to probe various ways in which inhibition could be important for speech perception. First, all target speech was presented in noise because it has been suggested that good inhibition is needed to reduce susceptibility to background noise (Janse, 2012). Second, target speech was varied in either (a) word frequency and neighborhood density for single words or (b) semantic context for sentences, because these lexical and semantic characteristics have been hypothesized to tax inhibition to different extents (Sommers and Danielson, 1999).

### Different scoring systems

*H1: If age-related changes in processing speed and sensory decline are independent contributors to Stroop scores in addition to inhibitory ability (Melara and Algom, 2003), we expect a low correlation between traditional scores (vSI*_raw_*), which do not take them into account, and the new scores (vSI*_res_*), which do*.

This hypothesis was assessed using the visual Stroop data. As shown in Table 12, correlations were extremely high between the vSI_raw_ and vSI_res_ measures. This suggests one of two possible interpretations: first, that the participants in this study had not experienced significant declines in color vision; or alternatively, that sensory decline and inhibitory ability were not independent processes. The first interpretation is unlikely given Ben-David and Schneider's (2009) meta-analysis, which strongly suggests that sensory decline amongst older people is widespread. The second interpretation implies that the two processes deteriorate in a comparable fashion, so that scores which account for sensory decline will nevertheless decline at a similar rate to those which do not. We think that this is a more likely explanation of our data.

*H2: We expect to see larger Stroop interference overall and greater variation in individual Stroop scores when examining slower trials*.

We investigated this hypothesis using the auditory Stroop data. Both of these hypotheses were supported by the data. To examine the slowest and fastest trials, we used normalized delta scores per quintile—that is, for each quintile of the RT distribution, we calculated Stroop interference effects and then normalized them according to the mean RT of the incongruent and neutral trials under examination. Despite using scores that were adjusted for overall RT, we nevertheless found the largest Stroop effects overall for Q5—the very slowest trials. We also found the widest range of Stroop scores in Q5, which implied that Stroop effects were not uniformly large in this quintile, but instead varied from being comparable to those in faster quintiles to being substantially increased. This supports the proposal of Ridderinkhof et al. (2004) that, although slower RTs allow for greater interference from a distractor, they also allow inhibition to build up and be deployed and, as a result, it is during these slowest responses that inhibitory differences become most apparent.

### Visual vs. auditory tasks

*H3: If inhibition is a modality-independent general cognitive ability, and if it influences individual performance to a greater extent than do task-specific demands, then the results from the visual and auditory Stroop tasks should be broadly comparable*.

Table 12 shows that the visual Stroop measures were entirely uncorrelated with the auditory Stroop measures; furthermore, the only correlation which neared significance—that of vSI_raw_ with aSI_ndeltaQ5_—was negative, meaning that the two measures in fact showed opposite trends. This was in stark contrast to within-task correlations, which showed that the two visual Stroop scoring systems and all four auditory Stroop scoring systems were closely correlated with each other. The only exception to this was the correlation between the aSI_ndeltaQ1_ and aSI_ndeltaQ5_ measures, which was moderate. This finding raises questions about the extent to which the two tasks measure the same aspect of cognition, either because separate inhibitory functions operate in different modalities or because task-specific demands outweighed the influence of inhibitory abilities in determining individual differences, or both.

### Relationship to SiN tasks

*H4: Larger Stroop interference scores are expected to be predictive of worse performance on SiN tasks, particularly when the SiN stimuli demand high levels of inhibition i.e., in less favorable SNRs, for isolated targets words, target words with low word frequency and/or high neighborhood density, or for low-predictability sentential context. These effects may be more pronounced for listeners with poorer hearing*.

We predicted a negative Stroop/SiN relationship, with larger Stroop effects predicting lower scores (i.e., worse performance) on SiN tasks. However, we only found this negative relationship in certain SiN conditions, and for certain listeners. For the auditory Stroop task, the overall direction of the relationship to SiN perception changed depending on which section of the RT distribution was under examination: for scores derived from the very slowest responses (aSI_ndeltaQ5_), the relationship was almost always positive; for scores derived from the very fastest responses (aSI_ndeltaQ1_), the relationship was generally negative—but even using these scores, some stimulus types, in conjunction with listener characteristics, produced a positive Stroop/SiN relationship. The fact that we found a negative Stroop/SiN relationship overall only when using the aSI_ndeltaQ1_ scores suggests that participants were engaged in two qualitatively different response modes: that for fast responses and that for slow responses, only the former of which was related to SiN perception in the predicted fashion. The reasons for this are unclear, but it is possible that participants were not always responding as fast as they could, despite instructions to do so. Delaying responses beyond the point at which the correct answer is accessed—for example, to mentally check the response, or because a regular rhythm of responding has been established—may distort Stroop effects in several ways. First, it may make it hard to distinguish between incongruent trials with failed (usually relatively longer RT) or successful (usually relatively shorter RT) inhibition, because responses to both are slow; second, if participants delay their responses to trials in the congruent condition, it may make Stroop effects appear smaller than they are, since it becomes harder to distinguish between trials with and without distractors. Distorted Stroop results are less likely to have a meaningful or interpretable relationship to SiN perception. In the case of the current data, if the fastest trials represent “true,” non-delayed responses while the slowest trials represent responses with an artificial delay, this may explain why the predicted Stroop/SiN relationship was seen only for the faster trials.

Assuming that the Stroop scores reliably reflected inhibitory abilities, we also expected the (negative) predictive effect of Stroop scores for SiN perception to interact with stimulus parameters such that a stronger effect was seen for those parameter levels which make listening harder and demand higher levels of inhibition. Specifically, these were the harder (as opposed to easier) SNR, isolated words as targets (as opposed to targets presented in sentences), low (as opposed to high) frequency and/or high (as opposed to low) neighborhood density targets, and/or targets in low (as opposed to high) predictability sentences. In some cases, we found this prediction to be true. For example, when using the vSI_res_ method, we found a stronger relationship between Stroop scores and word perception for high neighborhood density words than low neighborhood density words for those with poorer hearing sensitivity. However, the results are sometimes hard to interpret: for example, we found for many of the auditory Stroop scoring systems that the Stroop/SiN relationship was stronger at the less favorable SNR, and for two of these scoring systems the relationship was also stronger for words as opposed to sentences—but in these cases, the relationship was in the unexpected positive direction, and therefore did not indicate a greater predictive value in the expected sense. Finally, there were also cases in which the results ran directly against our hypothesis: for the vSI_raw_ scoring system, we found a stronger negative predictive Stroop/SiN relationship for words with low neighborhood densities, despite the fact that these words should theoretically demand a lower level of inhibition than their high neighborhood density counterparts. Similarly, when using the aSI_ndeltaQ1_ scoring system we found, for listeners with poor PTA, a stronger negative predictive Stroop/SiN relationship for sentences as opposed to words, despite the fact that isolated words should tax inhibition more than words presented within a sentential context. These results therefore suggest that, although the sentential context provides additional cues compared to the isolated words, these cues are not working in a consistent fashion to modulate the relationship between Stroop scores and SiN performance. Consequently, the questions of whether or not the Stroop scores genuinely provide a measure of inhibitory abilities, and whether inhibition is involved in SiN perception in a consistent manner, remain unanswered.

The suggestion that any effects might be particularly pronounced for those with poorer PTA scores was not generally borne out. There was a very limited role for PTA in the relationship between visual Stroop scores and SiN perception; this is perhaps to be expected given the non-auditory nature of the visual Stroop task. However, PTA played a similarly limited role when looking at the relationship between auditory Stroop scores and SiN perception; furthermore, the nature of those modulating effects which are present is unclear. The somewhat limited role of PTA in the results despite a large range of hearing sensitivity in the tested sample might be explained by the fact that stimuli were presented at 30 dB above each listener's individual SRT, which we hoped would to some extent mitigate difficulties caused by poorer hearing sensitivity.

*H5: If correlations between Stroop scores and SiN perception are driven by shared sensory decline, we expect the predictive power of Stroop interference for speech perception to decrease once sensory decline is accounted for. If the inhibition component drives the relationship, then a purer measure might improve the association between the two measures*.

For the visual Stroop task, the vSI_raw_ score appears to have slightly greater predictive value for SiN perception than the adjusted vSI_res_ score. As can be seen in Tables 3–5, models using the vSI_raw_ score almost always produce smaller AIC values (i.e., a better fit) than models using the vSI_res_ score. These differences were small, with AIC values for models using vSI_raw_ scores being only 1.74 smaller on average; however, this nevertheless suggests that the relationships between visual Stroop scores and SiN perception may rely in part on shared sensory decline. Without a measure of visual sensory decline, this hypothesis cannot be directly tested. At the very least, however, our findings suggest that taking sensory decline into account did not substantially enhance the predictive power of visual Stroop scores for modeling SiN perception in this case.

*H6: If Stroop scores derived from slower trials are better able to reveal individual differences in inhibitory ability, then these might be better predictors of SiN perception than average scores*.

For the auditory Stroop task, there was no evidence to suggest that the aSI_ndeltaQ5_ scoring system had greater predictive power for SiN perception than the other methods used. Indeed, as Tables 6–11 show, models using the aSI_ndeltaQ5_ scoring method consistently produced substantially larger AIC values (i.e., a poorer fit) than models using either the aSI_raw_ or aSI_norm_ methods. The average difference in AIC values between models using the aSI_ndeltaQ5_ scoring method and those using the aSI_raw_ and aSI_norm_ scores was 35.98 and 39.62 respectively.

## Conclusion

In this study we compared results from several different scoring systems for both visual and auditory Stroop tasks, and assessed their predictive value with respect to speech-in-noise perception. The results suggest that these two types of Stroop task may actually be measuring different aspects of cognition, rather than tapping a single modality-independent general cognitive ability. The use of different scoring systems changed the relationship of Stroop scores to speech-in-noise perception. On the one hand, this suggests that different scoring systems may allow different aspects of participants' responses to be selectively used in analysis—for example, isolating slower trials to measure the strongest inhibitory effects. However, it also suggests that traditional Stroop scores may not be reliable measures of inhibition, but may instead confound inhibitory abilities—or at least those abilities recruited in speech-in-noise perception—with task-specific demands and participant variables such as general response speed and visual acuity. Thus, caution must be exercised in the use of Stroop tasks and, if one is used, the scoring system must be carefully selected, particularly if there is any reason to suspect that participants may be experiencing age-related sensory declines or generalized slowing. Finally, hearing loss affected the relationship between Stroop scores and speech-in-noise perception, although inconsistently and only in some conditions. Nevertheless, the effects in these conditions highlight the importance of accounting for individual differences in both demographic factors and sensory acuity when analyzing cognitive data. Indeed, when choosing a cognitive task and/or scoring system, researchers may want to consider not just the nature of their outcome variable but also the degree to which they wish to minimize or emphasize the effects of listener variables.

## Limitations and further directions

It must be noted that there exists a range of cognitive functions referred to as “inhibition.” For example, Friedman and Miyake (2004) describe three inhibition-related functions:

1. Prepotent Response Inhibition (the ability to deliberately suppress a prepotent response, as tested in Stroop tasks)
2. Resistance to Distractor Interference (the ability to resist interference from irrelevant information in the external environment, as tested in e.g., flanker tasks)
3. Resistance to Proactive Interference (the ability to resist intrusions from memory of information that was previously task-relevant but is now irrelevant)

Using a variety of tasks to assess each function, they found that (1) and (2) were closely related, but neither was related to 3), suggesting at least two separate inhibitory functions of which the Stroop task probes only one. Furthermore, as noted above, no task is ever a “pure” measure of a given function, but always includes additional processes. In the current study, the Stroop task was chosen as the means of assessing inhibition because it is widely used in the literature, allowing us to directly compare our findings to those of other studies, and because of the questions it has raised surrounding cross-modal comparability and potential non-inhibitory confounds, allowing us to explore the ability of alternative scoring methods to address these issues. However, a different choice of task is likely to have tapped different inhibitory functions and/or different additional processes, and therefore produced different relationships both across task modalities and also with SiN perception. Nevertheless, this only confirms our view that any given “inhibition” task does not necessarily provide a reliable measure of general inhibitory abilities, and that care must be taken when selecting both tasks and scoring systems.

One important limitation of this study is its restricted pool of participants—we only tested older adults with mild hearing loss. Nevertheless, within these confines, participant variables had a considerable range: 25 years in age and 30 dB in hearing loss. This is important to keep in mind when examining data from other samples, since the range defines the potential size of the modulating effect. How the relationships found in this study generalize to other groups of listeners needs to be investigated in further work. The number of participants used in the study was also relatively small, which may mean that individual variability and/or measurement error obscured effects. Replication with larger sample sizes which show a greater variation in measures of interest is therefore desirable before firm conclusions are drawn.

It is also worth observing that the background masker used in the SiN task was speech-modulated noise, which contained no linguistic information. If the SiN stimuli had been presented in a speech masker, such as few-talker babble in which individual words were perceptible, then the observed relationships between SiN and Stroop scores might have been different. For example, it is possible that such SiN stimuli would demand a higher level of inhibition than those used here, since listeners would have to suppress not just noise but also lexical information, including the lexical neighborhood of masker words (Helfer and Jesse, 2015). However, it is hard to predict how this might have affected the Stroop/SiN relationship given the complex pattern of results obtained here. Finally, as discussed above, a further limitation of the study occurs in the form of the vSI_res_ measure, and in particular its reliance on a relationship based on the sample data rather than population norms. The predictive relationship between DI and Ci used to derive the vSI_res_ measure relies on the performance of the sample, which may not be representative of the wider population. If the vSI_res_ measure is considered to be useful, then future work should seek to establish an independent gold-standard relationship between Ci and DI.

## Author contributions

AH and SK designed the study and collected the data. SK analyzed the data. AH and SK interpreted the data. SK wrote, and AH contributed to, the manuscript and both contributed to the critical discussions. Both authors approved the final version of the manuscript for publication.

## Funding

This work was supported by the Medical Research Council (U135097128) and the Biotechnology and Biological Sciences Research Council (BB/K021508/1).

### Conflict of interest statement

The authors declare that the research was conducted in the absence of any commercial or financial relationships that could be construed as a potential conflict of interest.

## Footnotes

^1^First, the most complex model was run (i.e., full factorial: all main effects and all possible interactions). Then, non-significant effects were removed one level at a time. For example, if the highest-level interaction was a 4-way interaction and was not significant, it was removed. The model was subsequently re-run. All non-significant 3-way interactions were then removed, and the model was re-run. All non-significant 2-way interactions were then removed, and so on. If a previously significant higher-order interaction lost significance at any stage, this interaction was removed immediately before any further modifications were made. As a general rule, the principle of marginality was observed. As a consequence, if a higher-level interaction was kept in the model, the nested lower-level interactions were also retained. For example, if A × B × C was kept in the model, then the model also included A × B, A × C, and B × C. These relevant nested interactions are called “marginal effects”. As this approach has repercussions with regard to model parsimoniousness, a balance between the competing demands of marginality and parsimony was needed. This was achieved by keeping these guidelines in mind: (1) Even if the highest-level interaction was significant, it was not included in the model if it contained 5 or more factors. This allowed the models to be reasonably trimmed in the first instance. (2) A lower-level significant 5- or 4-way interaction was only kept in the model if it contained the Stroop variable. (3) All significant and/or marginal 3-way and 2-way interactions were included, regardless of whether they contained the Stroop variable. (4) All main effects were kept in the model at all times.

## Abbreviations

vSI_raw_: traditional Stroop interference score (visual)
aSI_raw_: traditional Stroop interference score (auditory)
Ci: overall color naming time, incongruent condition (visual)
Cn: overall color naming time, neutral condition (visual)
Rn: overall word reading time, reading condition (visual)
DI: dimensional imbalance (visual)
y_Ci_: observed Ci scores
ŷ_Ci_: predicted Ci scores
vSI_standard_: Ci scores standardized on DI (visual)
vSI_res_: residuals resulting from the difference between observed and predicted Ci scores (visual)
aRTi: average single-trial reaction time, incongruent condition (auditory)
aRTn: average single-trial reaction time, neutral condition (auditory)
aRTc: average single-trial reaction time, congruent condition (auditory)
vSI_norm_: normalized Stroop interference score (visual)
aSI_norm_: normalized Stroop interference score (auditory)
aSI_ndelta_: normalized delta score (auditory)
aSI_ndeltaQ5_: normalized delta score from slowest quintile of trials (auditory)
aSI_ndeltaQ1_: normalized delta score from fastest quintile of trials (auditory).
